# A novel therapy, using Ghrelin with pegylated G-CSF, inhibits brain hemorrhage from ionizing radiation or combined radiation injury

**DOI:** 10.15406/ppij.2019.07.00243

**Published:** 2019-06-26

**Authors:** JG Kiang, JT Smith, MN Anderson, MV Umali, C Ho, M Zhai, B Lin, S Jiang

**Affiliations:** 1 Radiation Combined Injury Program, Scientific Research Department, Armed Forces Radiobiology Research Institute, USA; 2 Department of Medicine, Uniformed Services University of the Health Sciences, USA; 3 Department of Pharmacology and Molecular Therapeutics, Uniformed Services University of the Health Sciences, USA; 4 Department of Biochemistry, University of California, USA

**Keywords:** animal model, mouse, Ghrelin, G-CSF, radiation, wound, brain, hemorrhage, platelet, ATP, NRF, AKT, MAPK, mdm2, p53, Caspase-3

## Abstract

Medical treatment becomes challenging when complicated injuries arise from secondary reactive metabolic and inflammatory products induced by initial acute ionizing radiation injury (RI) or when combined with subsequent trauma insult(s) (CI). With such detrimental effects on many organs, CI exacerbates the severity of primary injuries and decreases survival. Previously, in a novel study, we reported that ghrelin therapy significantly improved survival after CI. This study aimed to investigate whether brain hemorrhage induced by RI and CI could be inhibited by ghrelin therapy with pegylated G-CSF (i.e., Neulasta®, an FDA-approved drug). B6D2F1 female mice were exposed to 9.5 Gy ^60^Co-γ-radiation followed by 15% total-skin surface wound. Several endpoints were measured at several days. Brain hemorrhage and platelet depletion were observed in RI and CI mice. Brain hemorrhage severity was significantly higher in CI mice than in RI mice. Ghrelin therapy with pegylated G-CSF reduced the severity in brains of both RI and CI mice. RI and CI did not alter PARP and NF-κB but did significantly reduce PGC-1α and ghrelin receptors; the therapy, however, was able to partially recover ghrelin receptors. RI and CI significantly increased IL-6, KC, Eotaxin, G-CSF, MIP-2, MCP-1, MIP-1α, but significantly decreased IL-2, IL-9, IL-10, MIG, IFN-γ, and PDGF-bb; the therapy inhibited these changes. RI and CI significantly reduced platelet numbers, cellular ATP levels, NRF1/2, and AKT phosphorylation. The therapy significantly mitigated these CI-induced changes and reduced p53-mdm2 mediated caspase-3 activation. Our data are the first to support the view that Ghrelin therapy with pegylated G-CSF is potentially a novel therapy for treating brain hemorrhage after RI and CI.

## Introduction

Vast volumes of literature report that ionizing radiation (IR) produces detrimental, and potentially devastating, effects to cells, organs and systems in humans.^1–4^ These irradiated victims often also are subjected to other trauma such as wounds or burns. These combined radiation injuries (CIs) were observed at Hiroshima and Nagasaki, Japan, where 60–70% of victims received thermal burns concurrent with radiation injury.^1,2^ At the Chernobyl reactor meltdown, 10% of 237 victims exposed to radiation received thermal burns as well.^3^ Yet nowadays, public health concerns relating to radiation exposure are on the rise due to advanced development and proliferation of nuclear technologies, radiation and nuclear medicine, and nuclear weapon systems. The growing risk of radiation incidents from terrorist acts with mass casualties thereby warrants increased caution, attention, and study.

It is generally believed that radiation at any given dose affects biological systems, with each biological system succumbing to acute radiation syndrome (ARS) at certain specific “threshold” doses. The most radiosensitive organ is the first to show sickness–namely, bone marrow, where damage occurs within hours after total-body irradiation. Consequently, hematopoietic-ARS (H-ARS) results: low bone marrow cellularity and circulatory blood cell depletion present first; spleen shrinkage, followed by splenomegaly, results next; and by day 7, gastrointestinal tract (GI)-ARS arises. Ultimately, impairment of sensitive tissues that sustain crucial immunochemical and metabolic homeostasis, breach of biological barriers, and post-irradiation sepsis leads to multiple organ failure (MOF).^4–12^

Animal studies from literature clearly indicate that wounds,^13–15^ burns,^15–17^ sepsis,^18^ and/or hemorrhage^19^ aggravate ARS, particularly in CI. Similar observations were found in humans exposed to ionizing radiation and burn trauma.^20^ Moreover, radiation suppresses progenitor cells in wounded tissues and bone marrow, thereby leading to complications in tissue renewal, neovascularization for wound healing, as well as remodeling of microvascular beds.^21,22^ Therefore, it is essential to develop and identify countermeasure agents or combinations for managing CI. We previously investigated and reported beneficial effects of Ghrelin (a growth hormone-like peptide containing 28 amino acids), specifically amelioration of hematopoietic syndrome of ARS and recovery from CI-associated trauma in mice,^23^ including increased survival, mitigation of bodyweight loss, wound healing acceleration, as well as increased hematocrit values, neutrophil counts, lymphocyte counts, platelet counts, and bone-marrow cellularity.^23^ These results were the first to suggest that Ghrelin therapy effectively improved survival not only by attenuating CI-induced leukocytopenia, thrombocytopenia and bone-marrow damage but also by accelerating wound healing rate.^23^

Our laboratory recently reported that brain hemorrhage was observed on days 13–16 after irradiation in an experimental animal model of radiation combined with burn trauma.^24^ In that report, when mice were exposed to 15% total skin surface burn following 9.5 Gy ^60^Cobalt-γ photon radiation, extracranial hemorrhage and intracranial hemorrhage were found. Extracranial hemorrhage was observed in the olfactory lobe, mid-brain, and cerebellum. The latter displayed bleeding that was distributed widely. Histological examination showed subdural and intraparenchymal bleeding in the cerebral cortex and cerebellar cortex. Platelet depletion concurrently occurred, suggesting a correlation between platelet counts and brain hemorrhage.^24^ Radiation in combination with wound trauma causes cellular ATP depletion in the ileum, pancreas, brain, spleen, kidney, lung, and liver.^25^ In that report, we found that combined radiation with wound trauma induced cellular ATP reduction by inhibiting pyruvate dehydrogenase and activating pyruvate dehydrogenase kinase 1. A similar result was found in mice after hemorrhage.^26^

It is evident that CI increases MAPK activation.^27^ It was not clear whether CI alters AKT activation. Nevertheless, AKT and MAPK are known to be associated with apoptosis. Caspase-3 is a critical protease in caspase-dependent apoptosis.^28,29^ Pegylated G-CSF (Neulasta^®^) was approved by FDA in 2016 for hematopoietic syndrome of ARS.^30^ Pegylated G-CSF, which has been shown to significantly increase survival, modified hematological profiles after irradiation in our experimental animal model.^31,32^ Whether RI and CI would result in different severities of brain hemorrhage remained unclear. Furthermore, whether Ghrelin combined with pegylated G-CSF enabling to inhibit brain hemorrhage also remained unknown. In this report, we aim to investigate these two questions. Because CI is evident to amplify hematopoietic ARS and gastrointestinal ARS,^4^ we hypothesized that 1) CI results in greater brain hemorrhage than radiation alone, and 2) treatment with Ghrelin in the presence of pegylated G-CSF is effective in mitigating brain hemorrhage from RI and CI. Data presented in this report demonstrate that increases in brain hemorrhage incidents are associated with RI and CI and CI induced more lesions than RI. The increases are mitigated by Ghrelin therapy with pegylated G-CSF, thus proving the main hypotheses.

## Material and methods

### Animal and experimental design

All procedures involving animals were reviewed and approved by the AFRRI Institutional Animal Care and Use Committee. Euthanasia was carried out in accordance with the recommendations and guidance of the American Veterinary Medical Association. Research was conducted in a facility accredited by the Association for Assessment and Accreditation of Laboratory Animal Care (AAALAC).

B6D2F1/J female mice (10–12 weeks old, approximately 22–26 g) obtained from Jackson Laboratory (Bar Harbor, ME) were maintained in a facility accredited by AAALAC in plastic microisolator cages with hardwood chip bedding and allowed to acclimate to their surroundings for at least 7 days prior to initiation of the study. Male mice were not used in this study because of potential problems associated with male mouse aggression, such as fight wounds which were not desirable during the experimental period. Previous combined injury studies^23,24,31–33^ also used female mice for this reason. As such, we continued to conduct this study with female mice so that data collected could be compared with previous ones.

These mice were maintained in a facility accredited by the Association for Assessment and Accreditation of Laboratory Animal Care International in plastic microisolator cages on hardwood chip bedding. Commercial rodent chow (Rodent Diet #8604, Harlan Teklad, Madison, WI) and acidified tap water (pH=2.5–2.8) were provided *ad libitum*. Animal holding rooms were maintained at 22°C±2°C with 50%±20% relative humidity using at least 10–15 air changes/h of 100% conditioned fresh air. A 12-h 0600 (light) to 1800 (dark) full-spectrum lighting cycle was used. Mouse tails were tattooed for individual identification during acclimation. B6D2F1/J female mice were randomly divided into 8 groups:

- sham+vehicle+vehicle,
- wound+vehicle+vehicle,
- radiation+vehicle+vehicle,
- radiation+wound+vehicle+vehicle,
- sham+Ghrelin+pegylated G-CSF,
- wound+Ghrelin+pegylated G-CSF,
- Radiation+ Ghrelin+pegylated G-CSF, and
- Radiation+wound+Ghrelin+pegylated G-CSF.

Each group received topical gentamicin cream and was administered with oral levofloxacin. The sham-irradiated animals (equivalent to 0 Gy) were treated in the same manner but not exposed to the radiation source.

### Gamma irradiation

Mice were given 9.5 Gy^23,24,31–33^ whole-body bilateral ^60^Co γ-photon radiation, delivered at a dose rate of 0.4 Gy/min, as described previously.^23^ The dose of 9.5 Gy is the dose to cause 50% population death over 30 days postradiation, abbreviated LD_50/30_. The field was uniform within ±2%. The exposure time for each radiation was determined from the mapping data; corrections for the ^60^Co decay and the small difference in the mass energy absorption coefficients for water and soft tissue were applied. The accuracy of the actual dose delivery was verified with an ionization chamber adjacent to the mouse rack, which had been calibrated in terms of dose to the midline soft tissue of mice.

### Skin injury

Skin surface injuries were performed on the shaved dorsal surface of mice. Animals receiving skin wounds were anesthetized by isoflurane inhalation. A 15% total-body-surface-area skin wound was performed within 1h after irradiation.^23^ All mice subjected to the skin injury were given 0.5 mL sterile 0.9% NaCl intraperitoneally (i.p.), which contained 150mg/kg of acetaminophen (AmerisourceBergen, Glen Alen, Virginia) immediately after skin injury to alleviate pain. Four hours later, mice were given a second dose of 150mg/kg of acetaminophen. Skin-wounded mice without radiation exposure received only one dose of 150mg/kg of acetaminophen immediately after skin injury.

### Ghrelin administration

Ghrelin was purchased from Phoenix Pharmaceutical (Burlingame, CA). Three doses of 113μg/kg were administered subcutaneously (s.c.) in a volume of 0.2 mL 24h, 2d, and 3d after RI or CI. The vehicle given to control mice was sterile 0.9% sodium chloride solution for injection, USP, based on the survival data published previously.^23^

### Pegylated G-CSF administration

Pegylated G-CSF (Neulasta®; NDC: 555-13-019001) is a polyethylene glycol pharmaceutical-formulated-grade drug, also known as pegfilgrastim, that was purchased from the AmerisourceBergen Corporation (Valley Forge, PA). A dose of 1000μg/kg was administered by s.c. injections^31,32^ in a volume of 0.2ml 24 h, 8d, and 15d after RI or CI, i.e., 25μg/25-g mouse. Neulasta® was supplied in 0.6mL prefilled syringes for s.c. injection. Each syringe contains 6mg Peg-G-CSF in a sterile, clear, colorless, preservative-free solution containing 0.35mg acetate, 0.02mg polysorbate 20, 0.02mg sodium, and 30mg sorbitol in water for injection, USP. The vehicle mouse received 0.2ml of vehicle containing 0.35mg acetate, 0.02mg polysorbate 20, 0.02mg sodium, and 30mg sorbitol in 0.6mL water.^31,32^

### Antimicrobial agents

Gentamicin sulfate cream, 0.1% (generic, E. Fougera and Co., Melville, N.Y., NDC 0168-007-15), was applied daily for 10 days to the skin injuries on days 1–10. Levofloxacin (LVX), (generic, Hi-Tech Pharmacal Co., Inc., Amityville, NY, NDC 50383-286-04), 100mg/kg in 0.2mL/mouse, was administered p. o. daily for 14 days beginning on day 3.

### Platelet counts

Blood samples were collected in EDTA tubes after Sham, wound, RI and CI and assessed with the ADVIA 2120 Hematology System (Siemens, Deerfield, IL). Differential analysis was conducted using the peroxidase method and the light scattering techniques recommended by the manufacturer.

### Histopathology assessment

Mouse craniums and/or the extracted brains were kept in 10% neutral buffered formalin as above until processing by routine methods for histopathologic examinations. The formalin-fixed tissues were embedded in paraffin, cut into 5-μm sections, stained with hematoxylin and eosin, and examined by light microscopy. Histologic lesions were graded by number of hemorrhage lesions.

### Tissue lysates

Surviving mice were anesthetized by isoflurane followed by vertebrate dislocation on day 30 after sham, wound, RI and CI for blood collection and brain collection. Mice with moribundity were euthanized by CO_2_ during the 30-day monitoring period for collecting their blood and brains. Their entire brains from surviving mice and moribund mice were collected. Because the hemorrhagic lesions were dominant in small brain, the small brain was used for further biochemical studies. The small brains were mixed with Na^+^ Hanks’ solution containing 10μl/ml protease inhibitor cocktail, 10mM phosphatase 2 inhibitor, 10mM phosphatase 3 inhibitor, 10mM DTT, 5mM EDTA and 10mM PMSF, homogenized using Bullet Blender Homogenizer Storm (Next Advance, Averill Park, NY) for 4 min at speed 10 and centrifuged at 9,000 xg for 10 min (Sorvall Legend Micro 21 Centrifuge, Thermo Electron Corp, Madison, WI). Supernatant fluids were conserved for protein determination and stored at −80°C until use.

### Cytokine/chemokine measurements

Cytokine concentrations in small brain lysates were analyzed using the Bio-Plex^™^ 23 Cytokine Assay kit and 9 Cytokine kit (Bio-Rad Laboratories Inc., Hercules, CA) following the manufacturer’s protocol. Data were analyzed using the LuminexH 100^™^ System (Luminex Corp.; Austin, TX) and quantified using MiraiBio MasterPlexH CT and QT Software (Hitachi Software Engineering America Ltd.; San Francisco, CA). Data were expressed as pg/mg protein in tissues.

### Western blot

Total protein in the small brain lysates was determined with Bio-Rad reagent (Bio-Rad, Richmond, CA). Samples with 20μg of protein in Na^+^ Hanks’ buffer containing 1% sodium dodecyl sulfate (SDS) and 1% 2-mercaptoethanol were resolved on SDS-polyacrylamide slab gels (Novex precast 4–20% gel, Invitrogen, Carlsbad, CA). After electrophoresis, proteins were blotted onto a polyvinylidene difuoride (PVDF) membrane (0.45μm, Invitrogene) using a Tran-Blot Turbo System and the manufacturer’s protocol (Bio-Rad, Hercules, CA). The blot was then incubated for 90min at room temperature with 5% non-fat dried milk in tris-buffered saline-0.5% tween20 (TBST) at room temperature. After blocking, the blot was incubated with a selected antibody against PGC-1α, NRF1, NRF2, Mfn1, Total C1–5 Oxpho Rodent WB Antibody Cocktail (ABCAM, Cambridge, MA), Drp1 (Cell Signaling, Danver, MA), PARP (Invitrogen, Rockford, IL), Ghrelin receptors, GAPDH (Novus Biologicals, Littleton, CO), NF-κBp65, NF-κBp50, AKT, p-AKT, ERK1/2, p-ERK1/2, JNK. P-JNK, p38, p-p38 (Santa Cruz Biotechnology, Dallas, TX), and IgG (R & D Systems, Minneapolis, MN) at a final concentration of 1μg/ml in TBST-5% milk. The blot was washed 3 times (10 min each) in TBST before incubating for 60 min at room temperature with a 1000X dilution of species-specific IgG peroxidase conjugate (Santa Cruz, CA) in TBST. The blot was washed 6 times (5 min each) in TBST before detection of the peroxidase activity using the Enhanced Chemiluminescence kit (Amersham Life Science Products, Arlington Height, IL). IgG and GAPDH levels were not altered by radiation and used as a control for protein loading. Protein bands of interest were quantitated using the ImageJ program and normalized to either IgG or GAPDH levels. Data were expressed as intensity ratio to IgG or GAPDH levels.

### Measurements of cellular ATP Levels

Cellular ATP levels were determined using the ATP Bioluminescence Assay Kit HS II (Roche, Mannheim, Germany). Luminescence was measured with a TD-20/20 luminometer (Turner Designs, Sunnyvale, CA). Data were normalized to total protein and cellular ATP levels were expressed as fmol/μg protein.

### Statistical analysis

Data were expressed as mean±s.e.m. For each western blot and assay, the data were compared using the ANOVA, Tukey post hoc test, and student’s t-test with a significance level of 5%.

## Results

Radiation at 9.5 Gy was used to investigate the brain hemorrhage after RI and CI. This radiation dose is a lethal dose causing 50% population death within 30 days postirradiation (LD_50/30_) and has been used for previous publications on testing drug efficacy.^23,24,31–33^

### Ghrelin therapy with pegylated G-CSF mitigates hemorrhagic lesions on brain surfaces after irradiation or in combination with wound trauma

**Figure 1:**
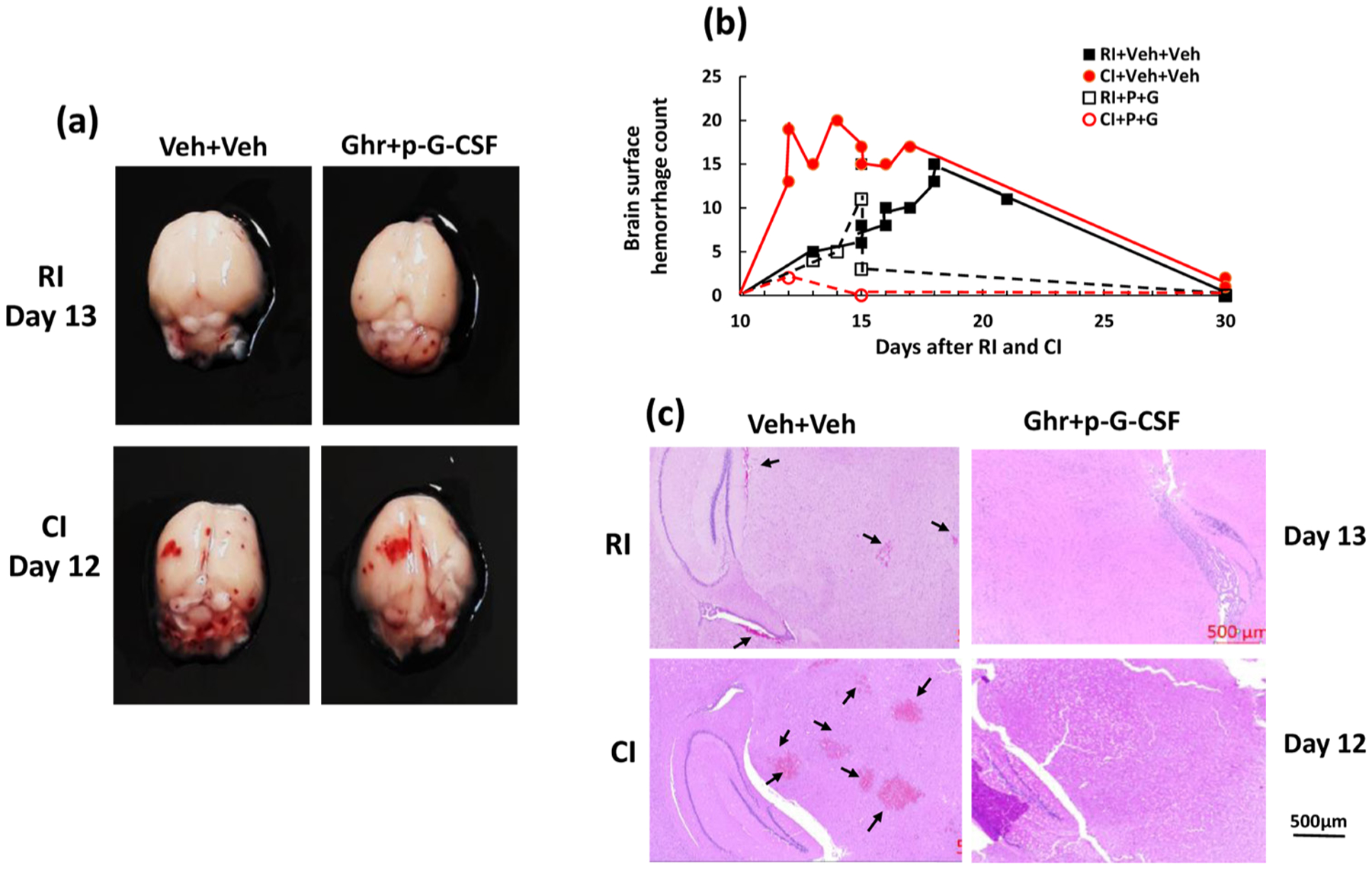
Ghrelin therapy with pegylated G-CSF inhibits brain hemorrhage after irradiation alone or in combination with wound. (a) Representative images with extracranial hemorrhage in brains of animals exposed to radiation alone (RI, moribund animal on day 13 post RI) or in combination with wound trauma (CI; moribund animal on day 12 post CI). (b) Quantitated analysis of hemorrhage counts on the brain surface of moribund mice. Each dot represents the number of hemorrhage lesions from each brain. (c) Representative images with intracranial hemorrhage in animals exposed to RI or CI. Black arrows indicate the hemorrhage lesions. RI, 9.5 Gy; CI, 9.5 Gy+wound; Veh, vehicle; Ghr or G, Ghrelin; P, pegylated G-CSF

Gross pathology assessments of skulls and brains obtained from B6D2F1/J mice revealed that all moribund animals from RI and CI experienced hemorrhages varying in extent, grade, hemorrhage type, and depth lesions. As shown in Figure 1a, brains collected from RI and CI mice displayed hemorrhage appearing in the cerebrum and cerebellum, with many hemorrhage lesions shown in the cerebellum. CI induced more hemorrhage lesions than RI (Figure 1b) at earlier time points. Gradually, brains from RI mice also reached similar quantities of hemorrhage lesions as CI. The brains of 30-day surviving mice showed no observable hemorrhage lesions after RI and 1 observable lesion after CI (data not shown). Ghrelin therapy with pegylated G-CSF significantly reduced hemorrhage lesions on the surface of cerebrum and cerebellum (Figure 1a–b).

### Ghrelin therapy with pegylated G-CSF mitigates hemorrhagic lesions in brains after irradiation or in combination with wound trauma

Our previous report showed the presence of hemorrhage in brains after irradiation followed by inflicted burn trauma.^24^ To evaluate the presence of intracranial hemorrhage after RI and CI herein, histological slides with H & E staining were made. As shown in Figure 1c, RI and CI induced intracranial bleeding lesions. CI induced more lesions than RI. Ghrelin therapy with pegylated G-CSF was effective in diminishing the lesions. As observed, massive deep confluent lesions occurred predominantly in the hindbrain, cerebellum, brain base, and olfactory bulbs, accompanied by subarachnoid hemorrhage in the structures as well (data not shown). Because hemorrhagic lesions were predominant in the cerebellum, cerebellum was collected for the following biochemical analysis including changes in cytokines, ATP, AKT and MAPK.

### Ghrelin therapy with pegylated G-CSF does not recover PGC-1α and NF-κB reduction in cerebellum after irradiation or in combination with wound trauma

**Figure 2:**
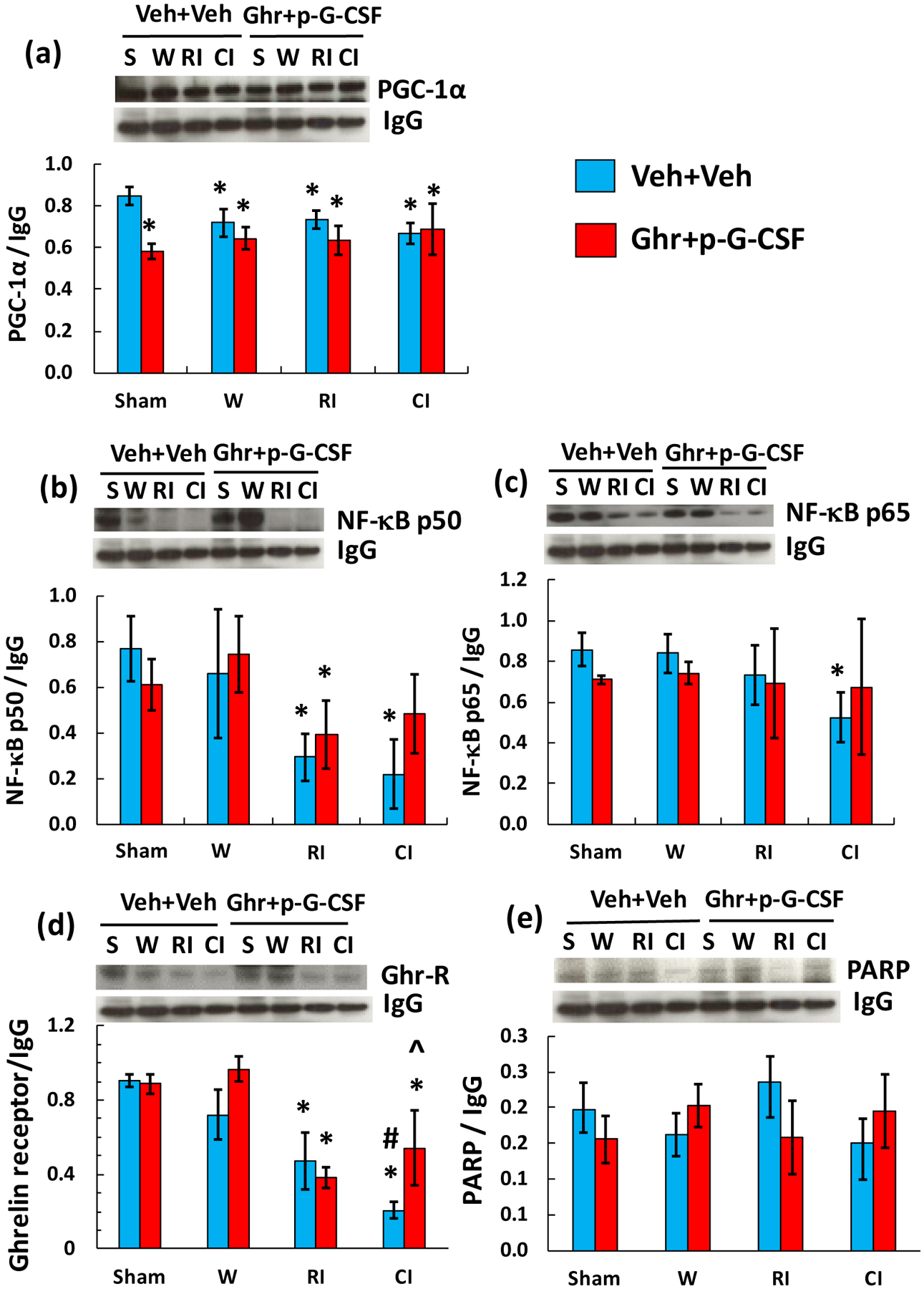
Ghrelin therapy with pegylated G-CSF does not alter RI or CI-induced decreases in PGC-1α (a), NF-κB (b and c), and PARP (e) but recovered ghrelin receptors (d). Representative gels for each group and quantitative protein bands of interest are presented. Data are presented as mean±sem. N=4 per group. *p<0.05 vs. Sham+Veh group. W, wound; RI, 9.5 Gy; CI, 9.5 Gy+wound; Veh, vehicle; Ghr, Ghrelin; p-G-CSF, pegylated G-CSF

Previously, we showed that RI and CI increased nuclear factor (NF)-κB activation in ileum and skin on days 1–7.^13^ The peroxisome proliferator-activated receptor (PPPAR)-γ coactivator-1α (PGC-1α) is shown to downregulate NF-κB levels.^34^ Therefore, we measured PGC-1*α* and NF-κB in the cerebellum through Western blotting analysis. RI and CI significantly reduced PGC-1*α* protein levels in cerebellum samples of mice treated with vehicle (Figure 2a). RI and CI significantly decreased NF-κB levels, because these are brain samples collected from moribund mice during a period between days 12–17 after RI and days 13–21 after CI. Ghrelin therapy with pegylated G-CSF did not improve the reductions (Figure 2b–c).

### Ghrelin therapy with pegylated G-CSF recovers ghrelin receptors but not PARP in cerebellum after irradiation or in combination with wound trauma

Ghrelin binds to ghrelin receptors that couple with G-protein and PLC to initiate the following cascade reactions in cells.^35^ It was of interest to find out whether RI and CI altered ghrelin receptors. Indeed, RI and CI decreased these receptors. Ghrelin therapy with pegylated G-CSF was able to recover these reductions in samples of CI mice (Figure 2d). The recovery was specific because RI and CI also decreased poly(ADP-ribose) polymerase (PARP, a protein to repair DNA double strand breaks), but the therapy failed to recover PARP (Figure 2e).

### Ghrelin therapy with pegylated G-CSF inhibits RI-induced increases in proinflammatory cytokines/chemokines in cerebellum

**Figure 3:**
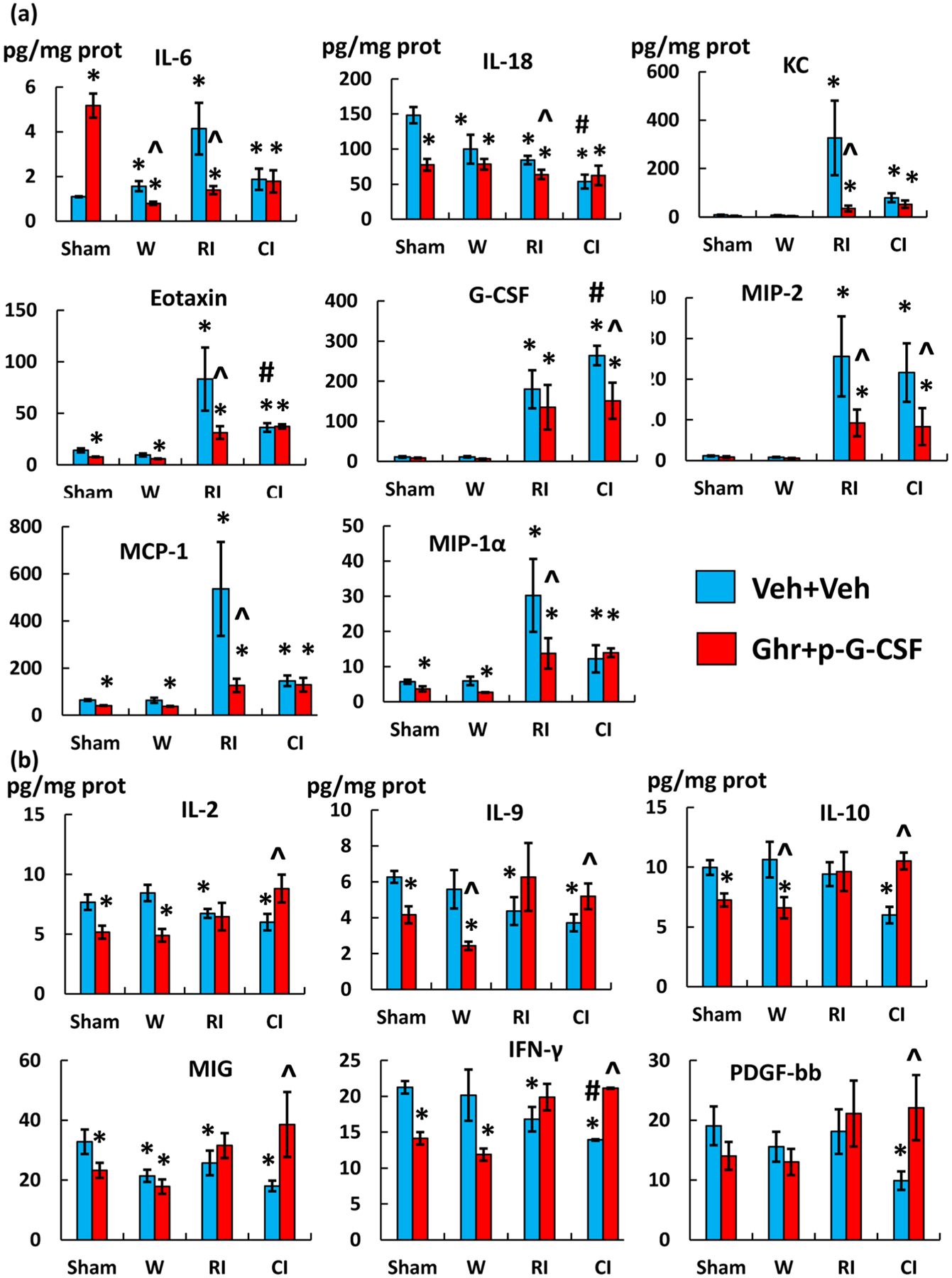
Ghrelin therapy with pegylated G-CSF modifies RI- and CI-induced changes in cytokine and chemokines in the small brain. Cytokines and chemokines were measured using multiplex kits. (a) Proinflammatory mediators. (b) Anti-inflammatory mediators. Data are presented as mean±sem. N=6 per group. *p<0.05 vs. sham+Veh group; #p<0.05 vs. RI+Veh group; ^p<0.05 vs. respective vehicle group. W, wound; RI, 9.5 Gy; CI, 9.5 Gy+wound; Veh, vehicle; Ghr, Ghrelin; p-G-CSF, pegylated G-CSF

RI increases cytokine/chemokines in blood and tissues.^19,23,33,36–38^ We found similar results in cerebellum after RI. RI increased IL-6, KC, Eotaxin, G-CSF, MIP-2, MCP-1, and MIP-1α, but decreased IL-18 (Figure 3a) in cerebellum lysates. Ghrelin therapy with pegylated G-CSF significantly mitigated these increases except for those associated with G-CSF (Figure 3a). CI amplifies increases in concentrations of cytokines/chemokines in blood and tissues.^19,23,33,36–38^ In cerebellum lysates, we found increases in IL-6, KC, Eotaxin, G-CSF, MIP-2, MCP-1, and MIP-1α, but decreases in IL-18 (Figure 3a). Ghrelin therapy with pegylated G-CSF did not mitigate these increases except G-CSF and MIP-2 (Figure 3a).

### Ghrelin therapy with pegylated G-CSF recovers CI-induced decreases in anti-Inflammatory cytokines/chemokines in cerebellum

RI decreased IL-2, IL-9, MIG, and IFN-γ (Figure 3b), whereas CI decreased IL-2, IL-9, IL-10, MIG, IFN-γ and PDGF-bb (Figure 3b). Ghrelin therapy with pegylated G-CSF significantly mitigated the IL-9 decrease, and fully recovered IL-2, IL-10, MIG, IFN-γ and PDGF-bb after CI, but not RI (Figure 3b).

### Ghrelin therapy with pegylated G-CSF partially recovers cellular ATP in cerebellum induced by radiation followed by wound trauma

**Figure 4:**
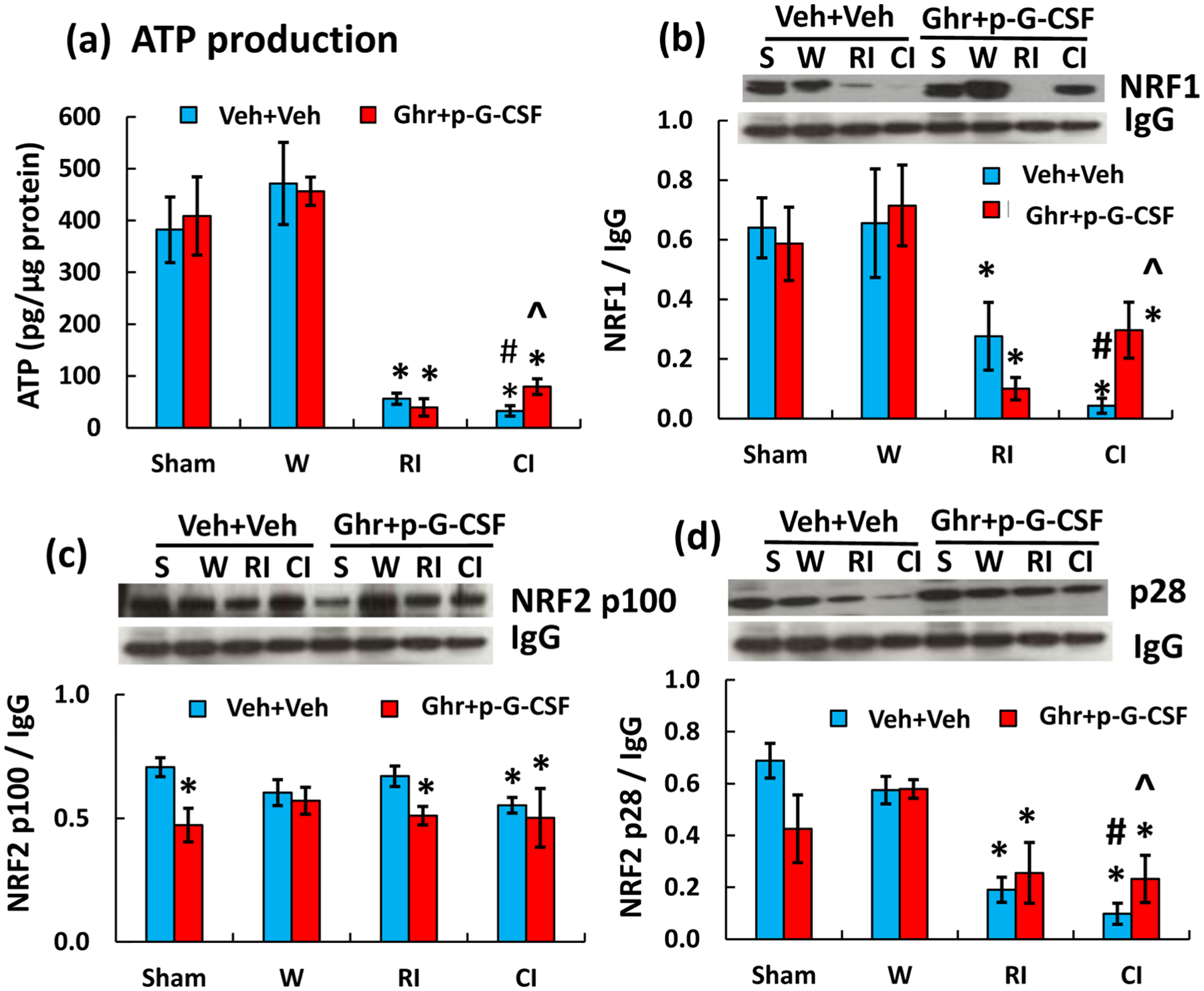
Ghrelin therapy with pegylated G-CSF modifies RI- and CI-induced decreases in ATP, NRF1 and NRP2 in the small brain. Representative gels and quantitated protein bands of interest were presented. Data are presented as mean±sem. N=4 per group. *p<0.05 vs. sham+Veh group; #p<0.05 vs. RI+Veh group; ^p<0.05 vs. CI+Veh group. W, wound; RI, 9.5 Gy; CI, 9.5 Gy+wound; Veh, vehicle; Ghr, Ghrelin; p-G-CSF, pegylated G-CSF

CI significantly reduces cellular ATP levels in many organs, including the whole brain.^25^ Herein, we found that RI and CI also remarkably reduced cellular ATP levels in the cerebellum (Figure 4a). Ghrelin therapy with pegylated G-CSF partially yet significantly recovered ATP levels only after CI (Figure 4a).

### Ghrelin therapy with pegylated G-CSF significantly recovers NRF1/2 in cerebellum induced by radiation followed by wound trauma

NRF1/2, important enzymes for ATP production,^39^ were found to be significantly reduced after RI and CI (Figure 4b–4d). CI reduced NRF1 (Figure 4b) and NRF2-p28 (Figure 4d) even more than RI. Ghrelin therapy with pegylated G-CSF partially but significantly recovered NRF1/2 levels only after CI (Figure 4b, 4d).

### Ghrelin therapy with pegylated G-CSF recovers complex III reductions in cerebellum induced by radiation combined with wound trauma

**Figure 5:**
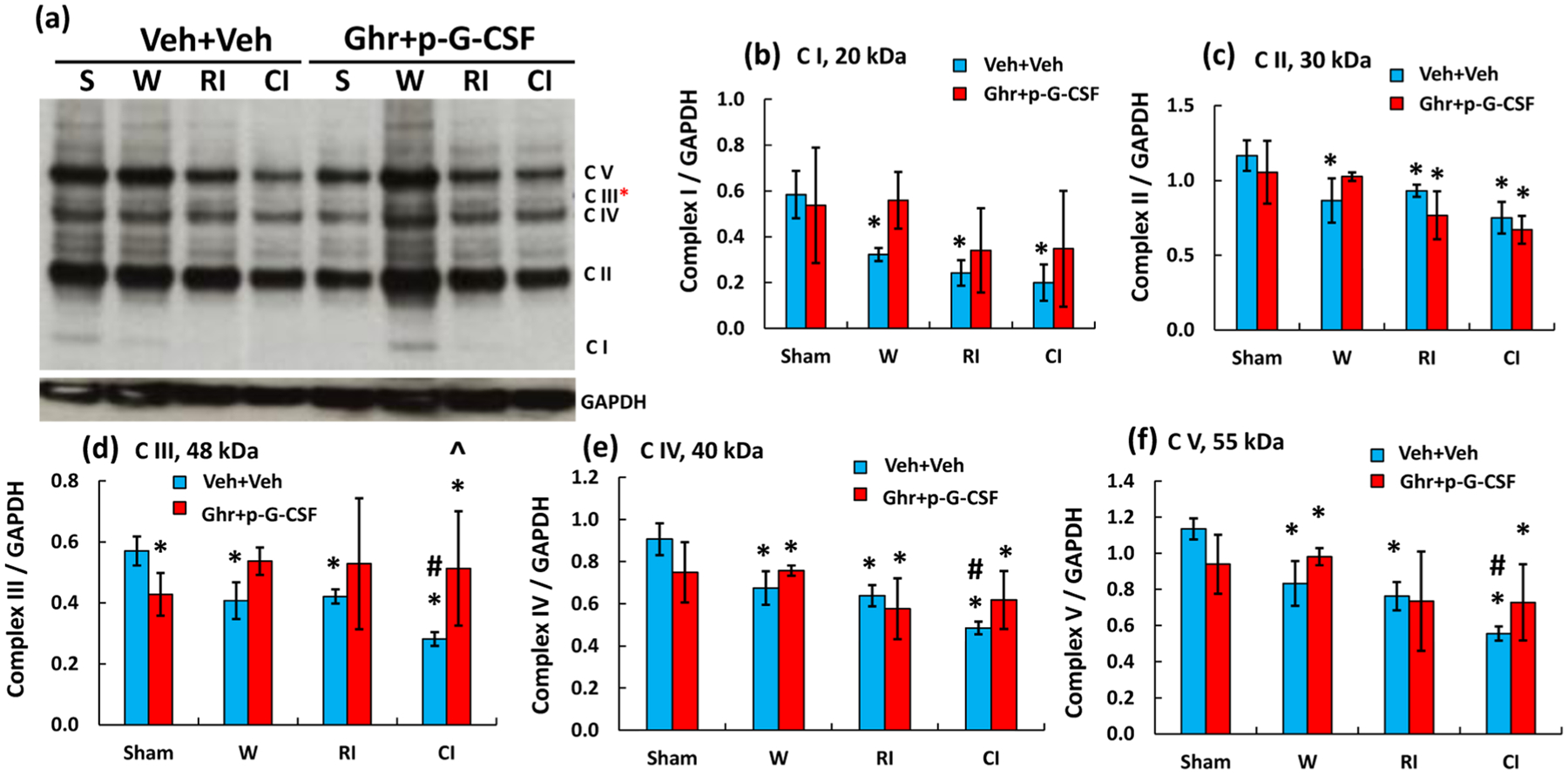
Ghrelin therapy with pegylated G-CSF recovers the CI-induced complex III in mitochondria of the small brain cells. (a) Representative gel image of complex I-V; (b-f) Quantitative complex I-V bands. Data are presented as mean±sem. N=4 per group. *p<0.05 vs. sham+Veh group; #p<0.05 vs. RI+Veh group; ^p<0.05 vs. CI+Veh group. W, wound; RI, 9.5 Gy; CI, 9.5 Gy+wound; Veh, vehicle; Ghr, Ghrelin; p-G-CSF or P, pegylated G-CSF

RI and CI also decreased complex I-V in electron transport chain in mitochondria using Western blot analysis. CI reduced complex III-V more than RI. Ghrelin therapy with pegylated G-CSF significantly recovered the complex III reduction (Figure 5).

### Ghrelin therapy with pegylated G-CSF decreases MAPK activation and increases AKT phosphorylation in cerebellum induced by radiation followed by wound trauma

**Figure 6:**
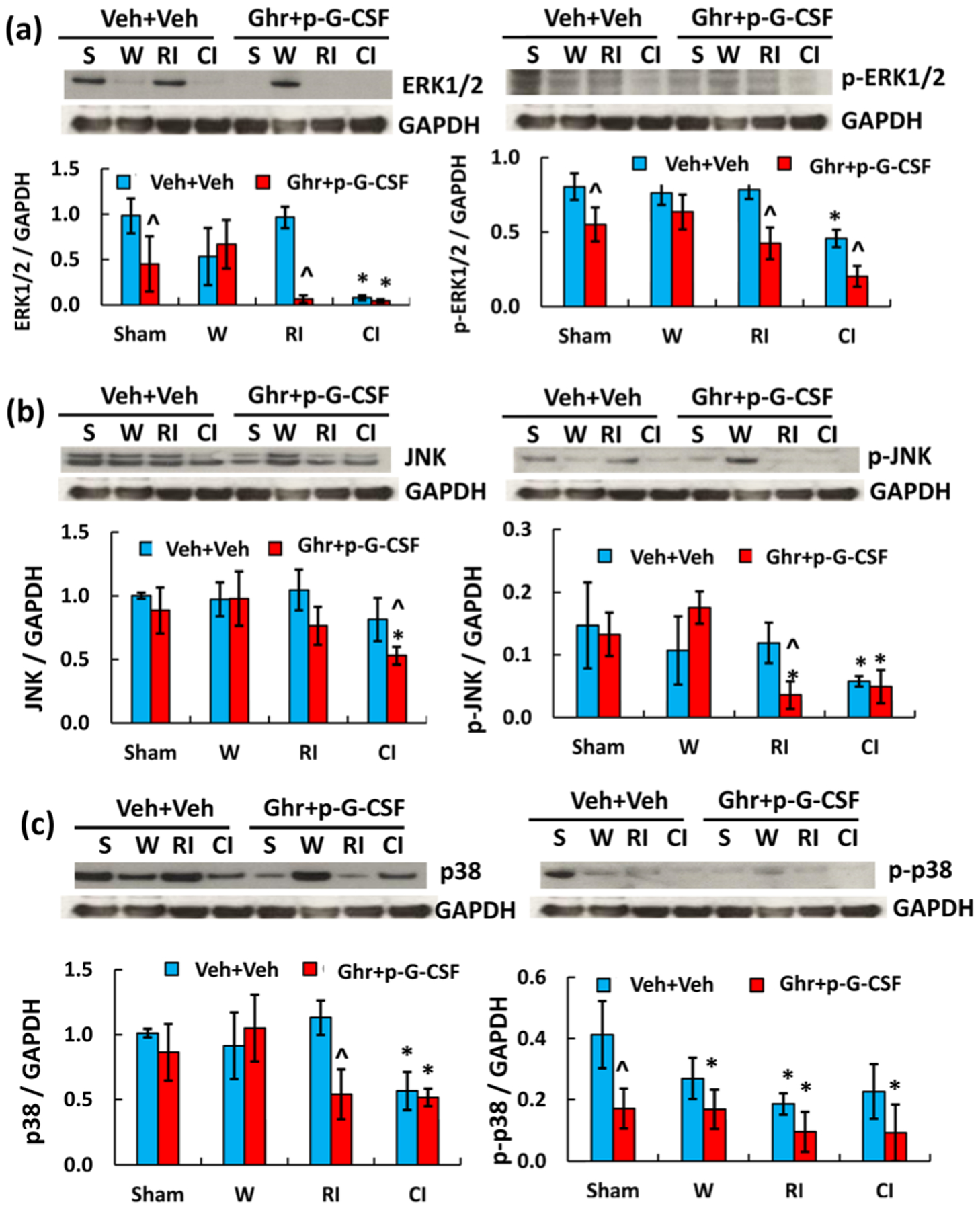
Ghrelin therapy with pegylated G-CSF modulates RI- but not CI-induced changes in MAPK activation in the small brain. (a) Representative gels and quantitated ERK1/2 and p-ERK1/2 bands; (b) Representative gels and quantitated JNK and p-JNK bands; (c) Representative gels and quantitated p38 and p-p38 bands, N=4 per group. Data are presented as mean±sem. *p<0.05 vs. sham+Veh group; #p<0.05 vs. RI+Veh group; ^p<0.05 vs. CI+Veh group. p-ERK1/2, phosphorylated ERK1/2; p-JNK, phosphorylated JNK; p-p38, phosphorylated p38; W, wound; RI, 9.5 Gy; CI, 9.5 Gy+wound; Veh, vehicle; Ghr, Ghrelin; p-G-CSF, pegylated G-CSF

**Figure 7:**
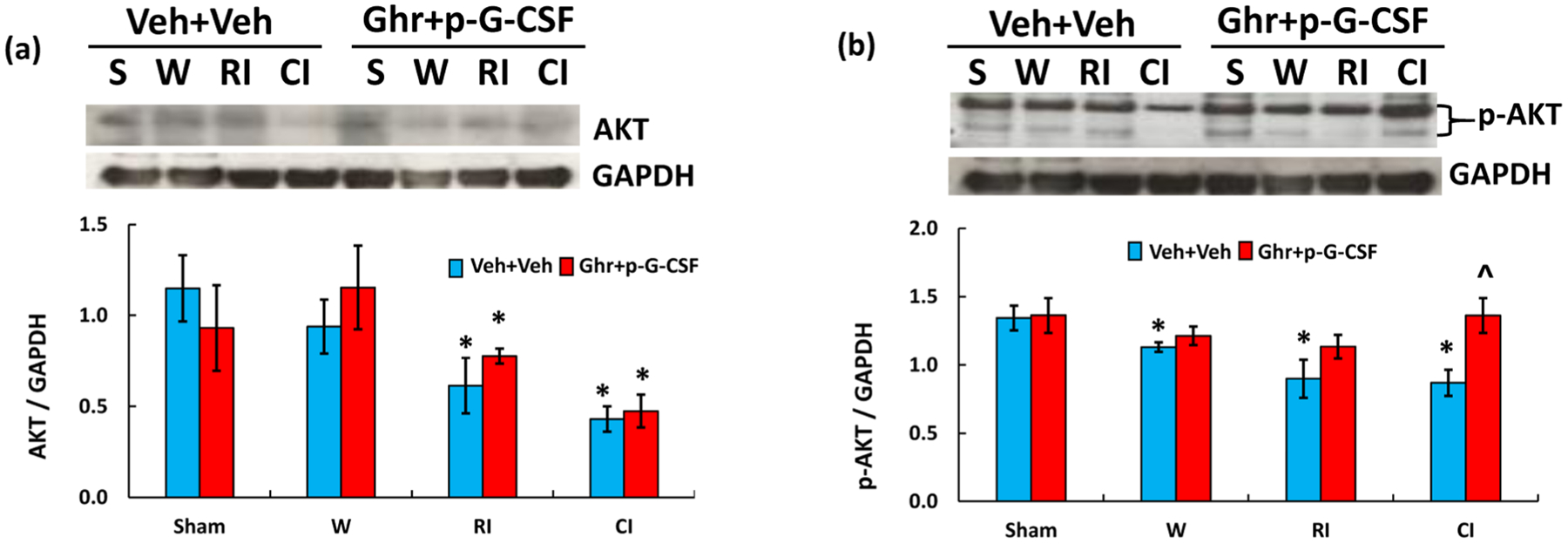
Ghrelin therapy with pegylated G-CSF recovers CI-induced decreases in AKT activation. (a) Representative gels and quantitated AKT bands were presented. (b) Representative gels and quantitated p-AKT bands were presented. N=4 per group. Data are presented as mean±sem. *p<0.05 vs. sham+Veh group; #p<0.05 vs. RI+Veh group; ^p<0.05 vs. CI+Veh group. p-AKT, phosphorylated AKT; W, wound; RI, 9.5 Gy; CI, 9.5 Gy+wound; Veh, vehicle; Ghr, Ghrelin; p-G-CSF, pegylated G-CSF

MAPK activation is observed after CI.^19^ As shown in Figure 6a, CI but not RI significantly reduced ERK1/2 and p-ERK1/2. Ghrelin with pegylated G-CSF further reduced p-ERK1/2 after RI and CI. In Figure 6b, CI and RI did not alter JNK but significantly reduced p-JNK. Ghrelin with pegylated G-CSF reduced p-JNK after RI but did not further reduce after CI. In Figure 6c, CI but not RI significantly reduced p38 and only RI significantly reduced p-p38. Ghrelin with pegylated G-CSF reduced p38 after RI but did not alter the RI-induced p-p38. RI and CI significantly reduced AKT (Figure 7a) and p-AKT (Figure 7b).^40–43^ Ghrelin therapy with pegylated G-CSF recovered appreciable amount of p-AKT after CI but not RI (Figure 7).

### Ghrelin therapy with pegylated G-CSF increases mdm2-p53 complex and decreases caspase-3 activation after irradiation and in combination with wound trauma

**Figure 8:**
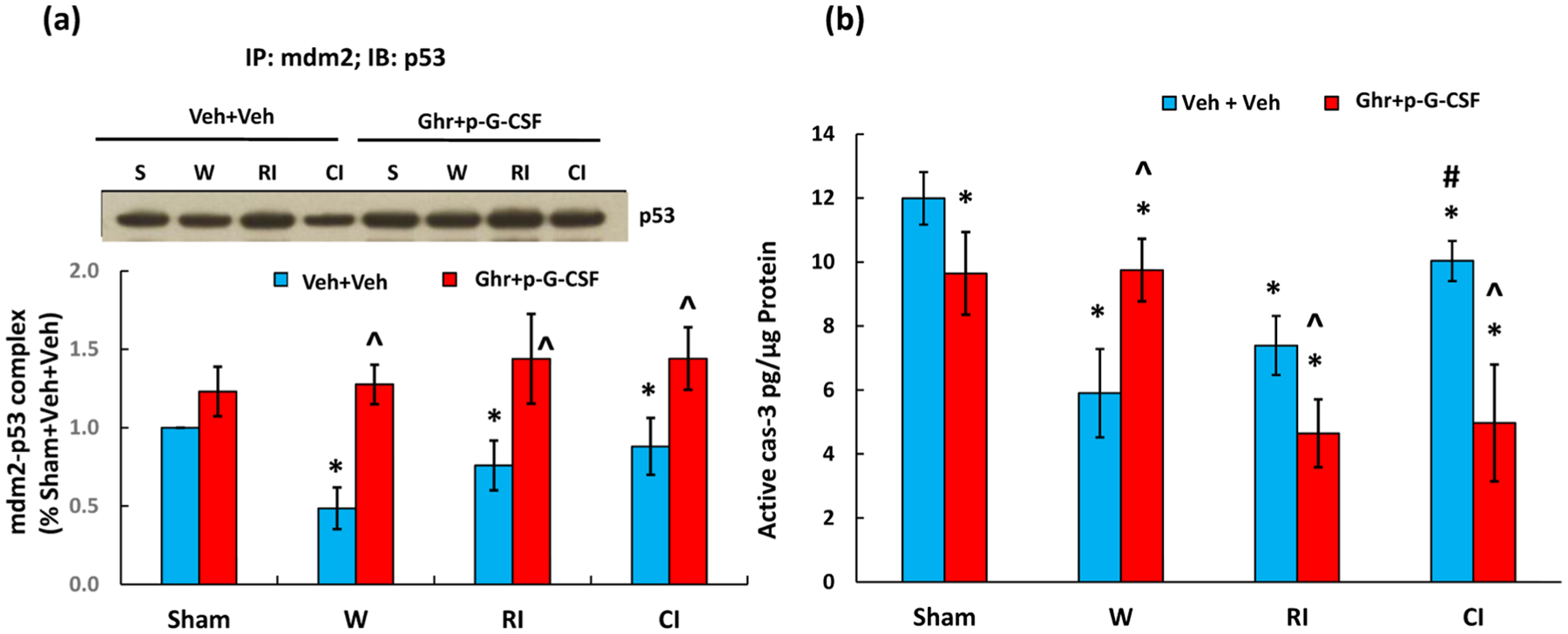
Ghrelin therapy with pegylated G-CSF upregulates mdm2-p53 complex and inhibits active caspase-3 activation. (a) Representative gel and quantitative mdm2-p53 complex band; (b) active caspase-3 levels, N=6–12 per group. Data are presented as mean±sem. *p<0.05 vs. sham+Veh group; #p<0.05 vs. RI+Veh group; ^p<0.05 vs. CI+Veh group. IP, immunoprecipitation; IB, immunoblotting; W, wound; RI, 9.5 Gy; CI, 9.5 Gy+wound; Veh, vehicle; Ghr, Ghrelin; p-G-CSF or P, pegylated G-CSF

AKT activation^44^ and NRF2 increases^45^ are known to reduce apoptosis. AKT stimulates MDM2 and inhibits p53.^4^ MDM2 conjugates with p53 to make less free p53 available for triggering apoptosis.^4^ Therefore, we measured mdm2-p53 complex using immunoprecipitation with mdm2 and immunoblotting against p53. Figure 8a shows that wounding, RI and CI significantly reduced the complex. Ghrelin therapy with pegylated G-CSF increased the complex, implying less free p53 available for initiating apoptosis. Caspase-3 activation (an apoptotic biomarker) was measured as well. Figure 8b shows that wound and RI and CI reduced active caspase-3 levels. Ghrelin therapy with pegylated G-CSF significantly decreased active caspase-3 in RI mice and CI mice.

### Ghrelin therapy with pegylated G-CSF mitigates platelet depletion caused by radiation

**Figure 9:**
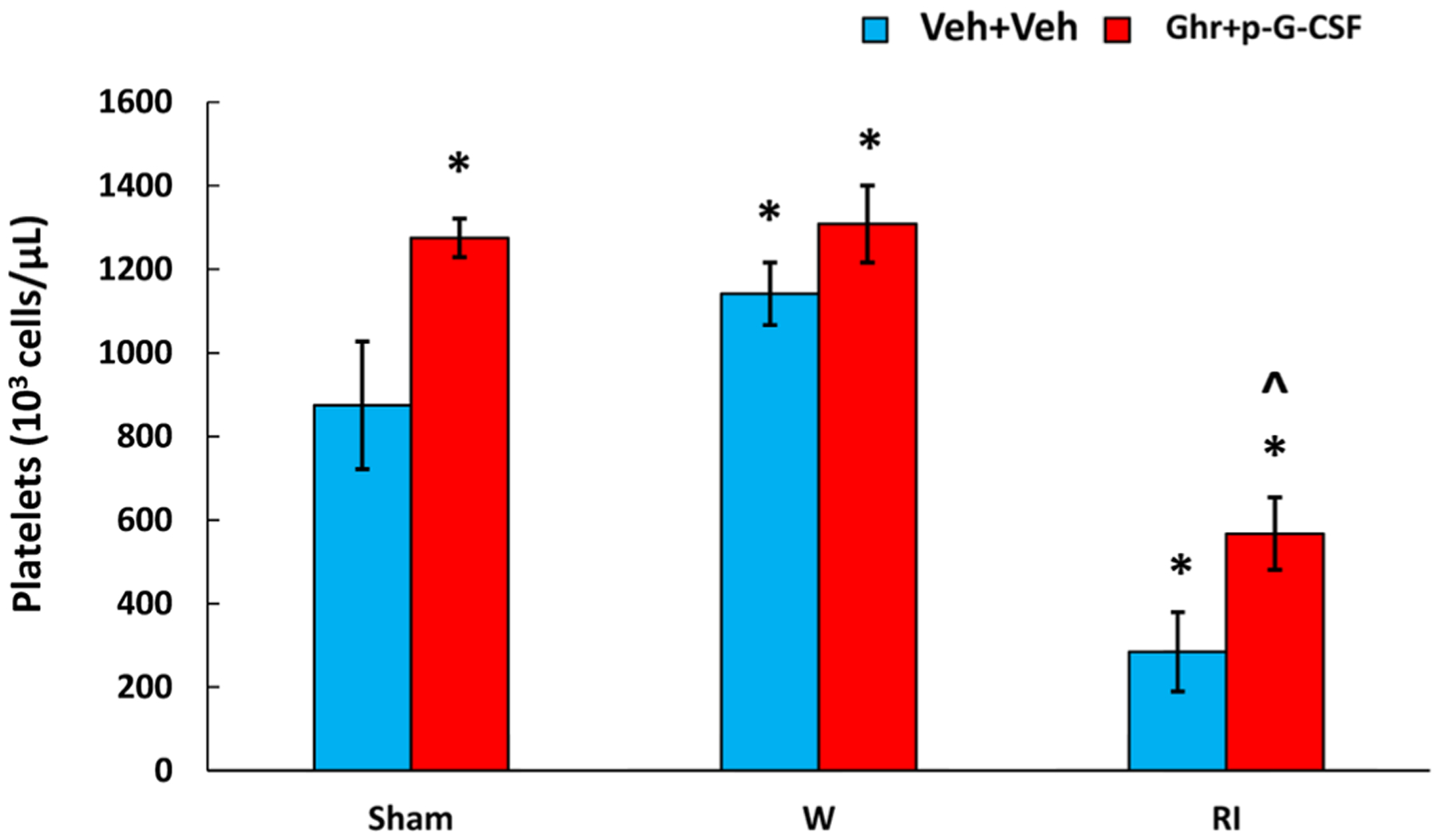
Ghrelin therapy with pegylated G-CSF recovers platelet counts after RI. N=6–12. Data are presented as mean±sem. *p<0.05 vs. sham+Veh group; ^p<0.05 vs. CI+Veh group. W, wound; RI, 9.5 Gy; Veh, vehicle; Ghr, Ghrelin; P, pegylated G-CSF

RI and CI are known to induce platelet depletion.^23^ As shown in Figure 9, in RI mice, platelets were counted. Platelet depletion was indeed observed (in 10^6^ cells/mL; sham+vehicle: 875±153 vs. RI+vehicle: 284±95; p<0.05). Ghrelin therapy with pegylated G-CSF mitigated this depletion and significantly elevated platelet counts back to 567±86 (p<0.05). No platelet counts were available in CI mice treated with the combinational therapy.

## Discussion

In this report, we provide evidence that in B6D2F1/J mice, brain hemorrhage is associated with RI- and CI-induced moribundity. CI induced brain hemorrhage earlier and more severely than RI. Ghrelin therapy with pegylated G-CSF after RI and CI mitigated sickness, moribundity and impact of brain hemorrhage. Either total or partial body radiation exposure results in damage of microvascular networks, which is one of the most important outcomes of acute radiation sickness.^9,10,21,46^ RI concurrently induces massive release of numerous reactive factors, coagulopathy, suppression of vascular growth factors, and vascular remodeling and complicates the endothelial injury-associated peripheral perfusion.^47,48^ The microvascular barriers (being composed of vascular endothelial cells, the basement membrane and pericytes) sustain circulatory homeostasis. Therefore, the impact of endothelium impairment becomes long-lasting from an acute phase to a delayed phase, and thereafter, to a prolonged phase.^2,21,47,48^ These effects of interstitial hemorrhage, cell hypoxia, and cell necrosis are life-threatening and represent a great challenge; not only in the development of countermeasures against radiological/nuclear accidents, but also can complicate outcomes in radiation therapy.^12,21,49,50^

RI and CI reduced cerebellar NF-κB, which was different from the previous observation in ileum and skin.^13^ The discrepancy is due to (1) different tissues analyzed and (2) the tissue collection at different time points after RI and CI. In this report, the brain of RI and CI mice were collected from moribund mice euthanized on days 13–21 and days 12–17, respectively. RI and CI reduced ghrelin receptors, PGC-1α and PARP in the cerebellum. Ghrelin administration with pegylated G-CSF partially recovered the receptors but not PGC-1α and PARP, suggesting that the therapy effect is specific. RI and CI increased proinflammatory cytokines and chemokines in the cerebellum. Ghrelin therapy with pegylated G-CSF effectively inhibited RI-induced proinflammatory cytokines and chemokines (Figure 3a) and increased anti-inflammatory cytokines and chemokines after CI (Figure 3b). The differential results from Ghrelin administration with pegylated G-CSF after RI and CI on cytokine/chemokines imply that RI and CI trigger different pathways to alter cytokines/chemokines in cerebellum.

We have previously reported that CI reduces cellular ATP levels,^25^ such that pyruvate dehydrogenase (PDH) is inactivated and pyruvate dehydrogenase kinase (PDK) is activated. In this report, we showed that RI and CI significantly reduced ATP production, with CI leading to more drastic reductions than RI in cerebellum. These reductions were mediated by decreased levels of NRF1/2. Like ATP, RI and CI also reduced NRF1/2, with CI leading to more drastic reductions than RI (Figure 4b, 4d). Similarly, mitochondrial complex III was reduced by CI more than RI (Figure 5a, 5d). However, Ghrelin administration with pegylated G-CSF partially recovered NRF1/2 and ATP but fully recovered complex III after CI. The correlation is apparent. This partial ATP recovery is important for CI mice to recover AKT phosphorylation that plays an important role in cell survival.

AKT activation is important for Ghrelin therapy with pegylated G-CSF mitigating CI. The increase in AKT phosphorylation may lead to inhibition of caspase-3 activation, a key player in caspase-dependent apoptosis and controlled by p53 which is regulated by mdm2. RI and CI decreased mdm2-p53 complexes, indicating more free form of p53 available to trigger apoptosis. The therapy increased mdm2-p53 complexes, suggesting less free form of p53 available. In contrast, Ghrelin therapy with pegylated G-CSF reduced p-ERK1/2 after RI and CI and p-JNK after RI, suggesting MAPK is also involved in this therapy. We have previously found increases in caspaspe-3 activation ubiquitously including kidney, heart, lung, brain, liver, and small intestine.^26^ Caspapse-3 mediated apoptosis found in cerebellum was inhibited by Ghrelin therapy with pegylated G-CSF (Figure 8b). The inhibition was mediated by reduced p53 availability (Figure 8a). The data are in agreement with observations in literature.^44,45^

The therapy was effective for RI mice as well, suggesting that a different mechanism appears to be involved. A possible mechanism underlying Ghrelin therapy with pegylated G-CSF in RI mice lies in platelet production. RI remarkably decreased platelet counts on day 7 and remained below the baseline even through day 30.^33^ Ghrelin therapy with pegylated G-CSF in RI mice increased platelets (Figure 9). Platelet sizes are about 2 μm in dimeter, whereas megakaryocyte sizes are about 100 μm in dimeter. Decreases in platelet counts stimulate thrombopoietin (TPO) production by the liver. Consequently, the number of megakaryocytes in bone marrow increases. The time required for megakaryocytes to complete polyploidization, mature, and release platelets is about 5 days in humans and 2–3 days in rodents.^51–53^ Once released into the bloodstream, human platelets survive 7–10 days, whereas rodent platelets in peripheral blood survive 4–5 days.^54–56^ The osteoblastic niche provides an environment that allows megakaryocytes to mature and develop, whereas the vascular niche enhances proplatelet formation.^57^ Therefore, the possibility of Ghrelin therapy with pegylated G-CSF stimulating the vascular niche cannot be excluded and should be further explored. Furthermore, Ghrelin has also demonstrated to sustain endothelial function and angiogenesis.^18,58–62^ It needs to bear in mind that acute inflammatory responses in both humans and rodents do not revert back to homeostasis but trigger a previously yet unappreciated consequence of immunological events that dictate subsequent immune response to infection.^63^ Whether Ghrelin therapy with pegylated G-CSF can reduce the consequence of immunological events are not known and worthy for exploration.

It is evident that radiation combined with burn trauma increases miR-690 and miR-223 in serum.^64^ Likewise, radiation combined with hemorrhage increases let-7e, miR-30e, and miR-29b;^19^ radiation combined with wound increases miR-34a (Kiang et al., unpublished data). The possibility of Ghrelin therapy with pegylated G-CSF modifies microRNAs that are associated with thrombopoiesis also cannot be excluded and should be further explored.

In summary, RI and CI significantly increased brain hemorrhage. CI induced more hemorrhage lesions than RI. These lesions were mitigated by Ghrelin treatment with pegylated G-CSF. RI and CI decreased ghrelin receptors, increased proinflammatory cytokines/chemokines, and decreased anti-inflammatory cytokines/chemokines in the small brain. Ghrelin therapy with pegylated G-CSF remarkably inhibited proinflammatory cytokines/chemokines in RI mice and elevated anti-inflammatory cytokines/chemokines in CI mice. RI and CI inhibited cellular ATP amounts by decreasing NRF1/2 and complex 1-V proteins. Ghrelin therapy with pegylated G-CSF recovered ATP, NRF1/2 and complex III in CI mice. In RI mice, the combinational therapy mitigated RI-induced platelet depletions, which may contribute to inhibition of brain hemorrhage. These results suggest that Ghrelin treatment with pegylated G-CSF is potentially useful for treating brain hemorrhage.

## Conclusion

**Figure 10:**
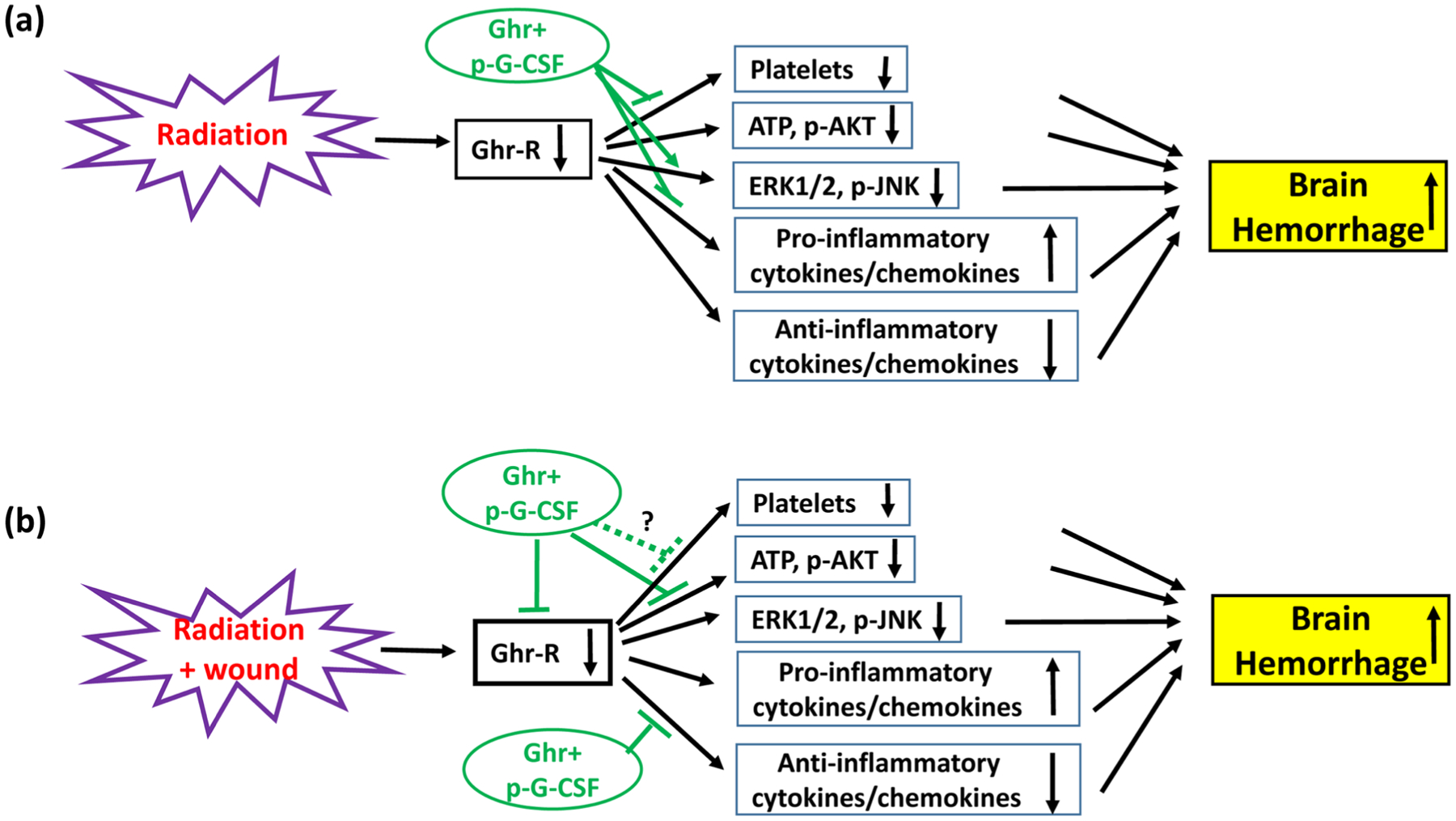
Schematic presentation of possible mechanisms underlying radiation alone or in combination with wound trauma. (a) Radiation reduces ghrelin receptors (Ghr-R), which may lead to decreases in platelets, cellular ATP production, anti-inflammatory cytokines/chemokines and phosphorylated AKT (p-AKT), as well as increases in pro-inflammatory cytokine/chemokines. As a result, these collectively lead to brain hemorrhage. Ghrelin therapy with pegylated G-CSF (Ghr+p-G-CSF) reduces brain hemorrhage lesions by recovering platelets, further inhibiting ERK1/2 and phosphorylated JNK (p-JNK), and blocking pro-inflammatory cytokines/chemokines. (b) Radiation combined with wound trauma displays a similar mechanism leading to brain hemorrhage. Ghrelin therapy with pegylated G-CSF reduces brain hemorrhage lesions by recovering Ghr-R, as well as increasing cellular ATP, p-AKT and anti-inflammatory cytokines/chemokines. ^⊥^, inhibition; ↓, decrease; ↑, increase

We demonstrate that ionizing radiation followed by skin wounds induces cerebro-vascular impairment, intracranial hemorrhage, ghrelin receptor reduction, cytokine/chemokine increases, cellular ATP reduction, and platelet depletion. The results suggest that ATP reduction and platelet depletion highly likely contribute to the onset of brain hemorrhage, at least in part; thereby, this intracranial hemorrhage partly leads to ultimate mortality. In RI mice, Ghrelin therapy with pegylated G-CSF significantly mitigated platelet depletion, proinflammatory cytokines/chemokines, and ERK1/2 and JNK activation (Figure 10a). In CI mice, it increased ghrelin receptors and anti-inflammatory cytokines/chemokines, while mitigating cellular ATP depletion, attenuating ERK1/2 and increasing AKT activation (Figure 10b). As a result, brain hemorrhage occurred. Taken together, Ghrelin therapy with pegylated G-CSF is a potentially effective therapy for RI and CI in reducing brain hemorrhage.

## Abbreviations:

AFRRI: Armed Forces Radiobiology Research Institute
AKT: serine/threonine-specific protein kinase
ARS: acute radiation syndrome
C I-V: complex I-V
CI: combined radiation injury
Drp1: dynamin-related protein 1
DTT: dithiothreitol
EDTA: ethylenediaminetetraacetic acid
H-ARS: hematopoiesis-acute radiation syndrome
IR: ionizing irradiation
Mfn1: mitofusin 1
NF-κB p50: nuclear factor-keppaB protein 50 kDa
NF-κB p65: nuclear factor-keppaB protein 65 kDa
NIH: National Institute of Health
NIAID: National Institute of Allergy and Infectious Diseases
IAA: Inter-agency agreement
NRF: nuclear respiratory factor
PARP: poly (ADP-ribose) polymerase
PGC-1α: peroxisome proliferator-activated receptor gamma coactivator 1-α
PMSF: phenylmethanesulfonyl fluoride
p.o.: *per os*
RI: radiation injury
s.c.: subcutaneously
